# Relative Validity of Micronutrient and Fiber Intake Assessed With Two New Interactive Meal- and Web-Based Food Frequency Questionnaires

**DOI:** 10.2196/jmir.2965

**Published:** 2014-02-21

**Authors:** Sara E Christensen, Elisabeth Möller, Stephanie E Bonn, Alexander Ploner, Olle Bälter, Lauren Lissner, Katarina Bälter

**Affiliations:** ^1^Karolinska InstitutetDepartment of Medical Epidemiology and BiostatisticsStockholmSweden; ^2^KTH Royal Institute of TechnologySchool of Computer Science and CommunicationStockholmSweden; ^3^University of GothenburgDepartment of Public Health and Community MedicineGöteborgSweden

**Keywords:** validity, reproducibility, FFQ, micronutrients, weighed food record, Internet, adult

## Abstract

**Background:**

The meal- and Web-based food frequency questionnaires, Meal-Q and MiniMeal-Q, were developed for cost-efficient assessment of dietary intake in epidemiological studies.

**Objective:**

The objective of this study was to evaluate the relative validity of micronutrient and fiber intake assessed with Meal-Q and MiniMeal-Q. The reproducibility of Meal-Q was also evaluated.

**Methods:**

A total of 163 volunteer men and women aged between 20 and 63 years were recruited from Stockholm County, Sweden. Assessment of micronutrient and fiber intake with the 174-item Meal-Q was compared to a Web-based 7-day weighed food record (WFR). Two administered Meal-Q questionnaires were compared for reproducibility. The 126-item MiniMeal-Q, developed after the validation study, was evaluated in a simulated validation by using truncated Meal-Q data.

**Results:**

The study population consisted of approximately 80% women (129/163) with a mean age of 33 years (SD 12) who were highly educated (130/163, 80% with >12 years of education) on average. Cross-classification of quartiles with the WFR placed 69% to 90% in the same/adjacent quartile for Meal-Q and 67% to 89% for MiniMeal-Q. Bland-Altman plots with the WFR and the questionnaires showed large variances and a trend of increasing underestimation with increasing intakes. Deattenuated and energy-adjusted Spearman rank correlations between the questionnaires and the WFR were in the range ρ=.25-.69, excluding sodium that was not statistically significant. Cross-classifications of quartiles of the 2 Meal-Q administrations placed 86% to 97% in the same/adjacent quartile. Intraclass correlation coefficients for energy-adjusted intakes were in the range of .50-.76.

**Conclusions:**

With the exception of sodium, this validation study demonstrates Meal-Q and MiniMeal-Q to be useful methods for ranking micronutrient and fiber intake in epidemiological studies with Web-based data collection.

## Introduction

The increasing use of the Internet worldwide [1] has made Web-based food frequency questionnaire (FFQ) methodology an attractive alternative to traditional paper-based instruments in epidemiological research. Today, more than 90% of the Swedish adult population has Internet access [2], which is a convincing rationale for choosing the Web over the paper-and-pencil method. Compared to paper-based FFQs, expenses are dramatically lower for Web-based versions for both dissemination and data handling, making it a more cost-efficient method [3,4]. In addition, the costs for the required software infrastructure have decreased over recent years [5]. The dynamic nature of the Web enables an interactive design with follow-up questions and skip patterns, which adapts the questions to the respondent’s answers thereby reducing the answering time. An interactive Web-questionnaire has previously shown high compliance in a Swedish population with widespread Internet access [6]. Taking advantage of the benefits of using the Web, we have developed 2 Web-based FFQs with an interactive design; Meal-Q and MiniMeal-Q. The questionnaires have a meal-based format to ease recall of food intake. This approach has shown promising results in previous studies when compared with traditional food group designs [7,8].

We have previously published results on the validity of energy and macronutrient intake assessed by Meal-Q and MiniMeal-Q with doubly labeled water and a weighed food record (WFR) as reference methods [9]. The present paper evaluates the validity of micronutrient and fiber intake assessed by Meal-Q and MiniMeal-Q by using the WFR as the reference method. We also present an evaluation of the reproducibility of Meal-Q.

## Methods

### Background

Meal-Q was developed with guidance from a population-based cross-sectional study on food products consumed for breakfast, lunch, dinner, and snack meals as reported by 700 randomly selected Swedish participants through either face-to-face interviews or 24-hour telephone recalls (E Möller and S Christensen, written communication, August 2008). In the spring of 2009, Meal-Q was evaluated in the VALidation of Methods Assessing diet and physical activity (VALMA) validation study. The reference method was a 7-day WFR on the Web. After the VALMA study was completed, we developed the shorter version, MiniMeal-Q, by omitting food items with low consumption frequency and low contribution to total energy and nutrient intake. However, food items that were important sources of specific nutrient intakes were kept (eg, black pudding, which contributes to iron intake). Moreover, varieties of similar food items were also kept to enable analyses of dietary patterns (eg, different types of bread and cereals with varying fiber and sugar content). By using truncated data from Meal-Q, we simulated a validity evaluation of MiniMeal-Q. Acknowledging that MiniMeal-Q originated from Meal-Q data, their validity comparison should be interpreted with caution.

### Recruitment

A total of 180 healthy volunteer men and women aged between 20 and 63 years were recruited through public announcement in Stockholm County, Sweden, to participate in the VALMA study. Announcements were made in the city, the suburbs, and at 2 universities, including among students in nutrition. Prerequisites for eligibility were access to the Internet and an email address, as well as not being on a weight-loss diet, nor being pregnant or having given birth during the past 10 months. All participants were informed about the study at an introductory meeting and gave their written informed consent. The Research Ethics committee at Karolinska Institutet approved the study.

### Study Design

**Figure 1:**
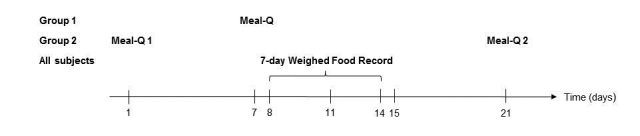
The 3-week study scheme of the VALidation of Methods Assessing diet and physical activity (VALMA) study. Data from the first administered Meal-Q from both groups was compared to the WFR for validity analyses. The same data from Meal-Q was truncated for simulated validity analysis of MiniMeal-Q. Meal-Q was distributed twice in group 2 for reproducibility analysis.

A study scheme of the 3-week validation study is shown in Figure 1. Participants were divided into 2 groups balanced for gender and age: group 1 (n=87) and group 2 (n=93). Group 1 filled out Meal-Q once, whereas group 2 also filled out a second Meal-Q after 3 weeks. Validity analysis with the WFR was made by using data from each participant’s first-administered Meal-Q. For the simulated validity analysis of MiniMeal-Q, data from the first Meal-Q from both groups was truncated and compared to the WFR. For reproducibility analysis, the first and second Meal-Q from group 2 were compared. The first-administered Meal-Q additionally included questions on education, occupation, and tobacco use (current smoking and Swedish snuff use). Each participant self-reported their height and weight, which were used to calculate body mass index (BMI, kg/m^2^).

### Dietary Assessment

#### Meal-Q

**Figure 2:**
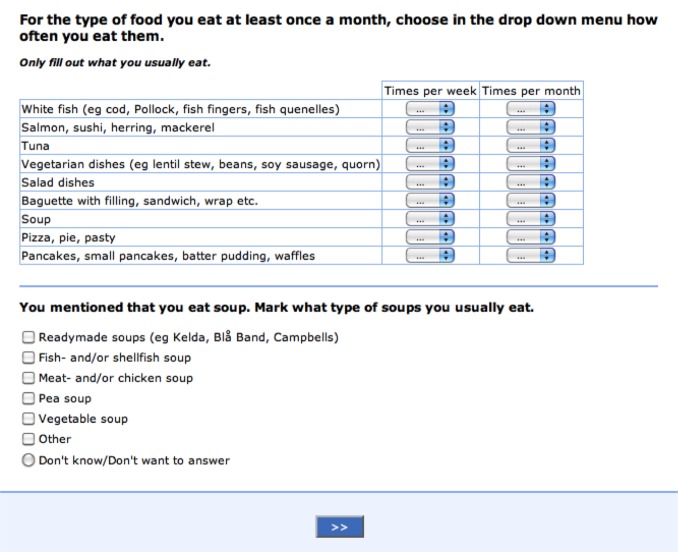
Screenshot of a Meal-Q module: lunch and dinner dishes and a follow-up question on soup. Translated from the Swedish questionnaire version in the VALidation of Methods Assessing diet and physical activity (VALMA) study.

Meal-Q is interactive and includes 102 to 174 food items depending on the number of follow-up questions (see Figure 2 for an example of a questionnaire module). The mean answering time was 17 minutes (SD 11) in the current study population [9]. The interactivity implies follow-up questions for high consumers of certain food items and dishes. Meal-Q assesses intake of food items, dishes, and beverages, which enables the calculation of energy and nutrient intake (including alcohol). It also asks about meal patterns; eating behavior, such as restaurant visits; intake of fast food, light products, probiotics, the use of cooking fat and salt, as well as the use of dietary supplements. Respondents choose among predefined food items and intake frequencies ranging from 1-3 times a month to 5+ times a day. Five photos of portion sizes are included for each of the following food groups: (1) rice/potatoes/pasta, (2) meat/chicken/fish/vegetarian alternatives, and (3) vegetables (raw or cooked). The photos are used to calculate portion sizes for cooked dishes and vegetables, whereas standard portion sizes are used for other food items. The standard portion sizes are derived from the National Food Agency, the Swedish Consumer Agency, measured portion sizes developed by the research group, and standard portion sizes used in other FFQs at Karolinska Institutet. For this validation study, Meal-Q asked about dietary intake during the past few months.

#### MiniMeal-Q

MiniMeal-Q contains 75 to 126 food items and is identical to Meal-Q in its design, including the interactive feature with adapted follow-up questions. The mean answering time for MiniMeal-Q was 7 minutes (SD 4) in a subsample of the current study population [9].

####  Weighed Food Record on the Web

At the introductory meeting, participants were given oral instructions and a handbook about how to fill out the 7-day WFR using a Web-based program, which covered more than 2000 food items. Each participant was given a kitchen scale and was asked to weigh and report all consumed food products and beverages at the highest level of detail possible. For example, a dish was encouraged to be reported by its individually weighed food items. As help for the recording throughout the day, all participants were provided with paper diaries. All records in the Web-based program were checked for completeness and reasonableness by the data collectors.

#### Nutrient Database

Daily intake of micronutrients and fiber was retrieved by linking intake of food items and dishes assessed with Meal-Q, MiniMeal-Q, and the WFR to the national database on nutrient content published by the Swedish National Food Agency [10]. The questionnaire’s nutrient conversion was made by computer programs (MealCalc and MiniMealCalc) developed and validated by the research group specifically for this study. The nutrient conversion of the food items and dishes assessed with the WFR was built into the Web-based WFR program. The nutrient conversions did not include dietary supplements.

### Assessment of Physical Activity Level for Identification of Energy Underreporters

A 7-day pedometer-based physical activity record provided in conjunction with the WFR program was filled out by all participants. The information was used to calculate the physical activity level (PAL) for each participant. Individual PAL values were also obtained from measurements of energy expenditure by the doubly labeled water (DLW) method [11] for 39 participants in group 2. A detailed description of the use of the DLW method in the VALMA study has been published previously [9,12]. The PAL values derived from the pedometers and from the DLW measurements were used for identification of potential energy underreporters in the WFR to exclude them from the comparison with Meal-Q and MiniMeal-Q.

### Statistical Analysis

Descriptive characteristics of the study participants are presented as mean (SD) and as counts (%). A 2-sample *t* test was used to assess differences in BMI and age between study groups, between men and women, and between included and excluded participants. Fisher’s exact test was used to assess differences in education, nutrition background (studying or working in the nutrition field), and tobacco use. All tests were 2-sided with significance level alpha=.05.

Median (interquartile range, IQR) crude micronutrient and fiber intake was calculated and compared between Meal-Q, MiniMeal-Q, and the WFR, and differences between the methods were determined by using Wilcoxon signed rank tests. Linear regression was used to calculate the between-person variance captured in the truncated MiniMeal-Q as compared to Meal-Q. Identification of energy underreporters was made by using the Goldberg cut-off method [13]. The cut-off was calculated by using the energy intake from the WFR together with the obtained PAL values from the physical activity record and the DLW data.

For validity and reproducibility analyses, micronutrient and fiber intakes were adjusted for total energy intake by using the residual method [14]. To test the ranking agreement and magnitude of misclassification of the questionnaires in comparison to the WFR, we used quartile cross-classifications, calculating proportions of participants classified into the same, adjacent, and extreme quartiles of energy-adjusted intakes. Bland-Altman plots were presented for Meal-Q, MiniMeal-Q, and the WFR to evaluate absolute agreement and differences in bias within the intake range [15]. The differences between the questionnaires and the WFR were plotted against the mean of the 2 methods and the degree of variation was represented by the limits of agreement (ie, ±2 SDs of the mean difference). Most variables were not normally distributed after energy adjustments; therefore, Spearman rank correlation coefficients were used to compare the questionnaires to the WFR. Deattenuated correlations corrected for within-person variation in the WFR were calculated by using the formula of Beaton et al [16] and Liu et al [17] and confidence intervals were produced by using the method of Willett and Rosner [18].

Reproducibility of Meal-Q was evaluated by comparing crude median (IQR) micronutrient and fiber intake between the first and second Meal-Q and by cross-classification of energy-adjusted [14] quartiles. Intraclass correlation coefficients (ICCs) [19] were also computed by using 1-way ANOVA with random effects.

Statistical analyses were performed using STATA statistical software version 11.2 (StataCorp LP, College Station, TX, USA).

## Results

### Exclusions

One participant was excluded because of dropout (group 1) and 2 because of illness (group 2). With the Goldberg cut-off method, 14 participants (4 in group 1, 10 in group 2) were identified as energy underreporters in the WFR and were excluded. Hence, 163 participants remained for validity analysis (group 1: n=82; group 2: n=81). We found no significant differences between included and excluded participants in terms of age, BMI, education, nutrition background, or tobacco use (*P*=.16-.99). In the WFR assessments, 1 participant had implausibly high intakes of beta-carotene (>30,000 µg/day) and 3 other participants had implausibly high intakes of sodium (>9000 mg/day). Therefore, they were excluded in each respective nutrient analysis. For reproducibility analysis of Meal-Q, no exclusion of energy underreporters were made; however, 4 participants had missing values in the second administered Meal-Q, leaving 87 participants in the analysis.

### Descriptive Statistics

General characteristics of the study participants included in the validity analysis are shown in Table 1. Most participants were highly educated (130/163, 80%) or students (95/163, 58%), one-third (54/163, 33%) were working full time, nearly one-third (49/163, 30%) had a nutrition background, and few participants (21/163, 13%) used tobacco. There were no statistically significant differences between study groups or sexes regarding age, BMI, education, nutrition background, smoking, or multivitamin/mineral supplement use (*P*=.05-.99). However, more men than women used Swedish snuff (*P*=.001). The between-person variance in micronutrient and fiber intake captured by MiniMeal-Q as compared to Meal-Q was 70% to 100%.

**Table 1:** Characteristics^a^ of participants included in the validity analysis (N=163).

Characteristics		Group 1 (n=82)	Group 2 (n=81)	Men (n=34)	Women (n=129)	All (N=163)
**Sex, n (%)**						
	Male	16 (20)	18 (22)			
	Female	66 (80)	63 (78)			
Age (years), mean (SD)		34 (12)	32 (11)	33 (10)	33 (12)	33 (12)
BMI (kg/m^2^), mean (SD)		23 (4)	23 (4)	24 (2)	23 (4)	23 (4)
Education >12 years, n (%)		64 (78)	66 (81)	27 (79)	103 (80)	130 (79.8)
Working full time, n (%)		33 (40)	21 (26)	12 (35)	42 (33)	54 (33.1)
Student, n (%)		41 (50)	54 (67)	18 (53)	77 (60)	95 (58.3)
Nutrition background,^b^ n (%)		21 (26)	28 (35)	6 (18)	43 (33)	49 (30.1)
Tobacco use,^c^ n (%)		11 (13)	10 (12)	12 (35)	9 (7)	21 (12.9)
Multivitamin/mineral supplement used, n (%)		18 (22)	14 (17)	8 (24)	24 (19)	32 (19.6)

^a^There was no statistically significant difference in characteristics between groups or sexes (*P*=.05-.99), except for Swedish snuff between sexes (1.8% women and 4.2% men, *P*=.001) using 2-sample *t* test and Fisher’s exact test.

^b^Studying or working in the nutrition field.

^c^Tobacco use=current smoking and/or Swedish snuff use. Values are missing for 3 women in group 2.

^d^Daily or weekly supplement use assessed with Meal-Q.

### Validity

The median (IQR) intake of most nutrients was higher when assessed with the WFR than with Meal-Q and MiniMeal-Q (Table 2). Exceptions were beta-carotene intake, which was higher when assessed with Meal-Q, whereas the intake was comparable between the WFR and MiniMeal-Q. There were no differences between the WFR and Meal-Q for thiamine, folate, magnesium, and fiber intake. Nor were there any differences between Meal-Q and MiniMeal-Q regarding any of the nutrients.

Quartile cross-classifications of micronutrient and fiber intake with the WFR and the questionnaires (Table 3) placed 69% to 90% of the participants into the same or adjacent quartile for Meal-Q, with the highest-ranking agreement for fiber and the lowest for sodium. For MiniMeal-Q, the ranking agreement ranged from 67% to 89%, also with fiber having the highest and sodium the lowest agreement. Proportions of participants in the extreme quartile ranged from 1% to 10% for Meal-Q and from 3% to 11% for MiniMeal-Q; the lowest proportions were found for vitamin C, magnesium, and fiber, and the highest for sodium.

**Table 2:** Median (IQR) daily crude micronutrient and fiber intake^a^ assessed with the weighed food record (WFR), Meal-Q, and MiniMeal-Q (N=163).

Nutrients	WFR, median (IQR)	Meal-Q, median (IQR)	MiniMeal-Q, median (IQR)
Beta-carotene (µg)	2632 (2539)^b^	3372 (2905)	3254 (3079)
Thiamine (mg)	1.5 (0.5)	1.4 (0.8)	1.3 (0.8)
Riboflavin (mg)	1.9 (0.7)	1.7 (0.9)	1.5 (0.8)
Niacin (mg)	36 (14)	15 (9)	14 (8)
Vitamin B_6_ (mg)	2.3 (1.0)	1.8 (1.0)	1.7 (0.9)
Folate (µg)	334 (167)	315 (210)	289 (193)
Vitamin B_12_ (µg)	5.7 (3.7)	3.8 (2.4)	3.5 (2.3)
Vitamin C (mg)	121 (92)	101 (82)	94 (74)
Vitamin D (µg)	5.6 (4.0)	4.7 (3.3)	4.4 (3.1)
Vitamin E (mg)	11 (5)	10 (5)	9 (5)
Calcium (mg)	1052 (381)	897 (583)	828 (512)
Iron (mg)	13 (6)	13 (7)	11 (6)
Magnesium (mg)	413 (177)	397 (242)	358 (207)
Phosphorus (mg)	1570 (514)	1433 (731)	1305 (677)
Potassium (mg)	3437 (1332)	3130 (1600)	2837 (1477)
Selenium (µg)	45 (21)	44 (24)	36 (22)
Sodium (mg)	3194 (1212)^c^	2448 (1118)	2158 (1015)
Zinc (mg)	12 (4)	11 (5)	10 (5)
Fiber (g)	25 (15)	26 (20)	23 (18)

^a^Most nutrient intakes assessed with the WFR were higher than intakes assessed with Meal-Q and MiniMeal-Q (*P*<.001-.03). Exceptions were beta-carotene intake, which was assessed higher with Meal-Q (*P*=.03), but was similar comparing the WFR and MiniMeal-Q (*P*=.19). Thiamine, folate, magnesium, and fiber intake were similar between the WFR and Meal-Q (*P*=.16-.92). There was no difference in intakes between Meal-Q and MiniMeal-Q (*P*<.001). (Wilcoxon signed rank test).

^b^n=162 because of exclusion of 1 participant with implausibly high intake.

^c^n=160 because of exclusion of 3 participants with implausibly high intakes.

**Table 3:** Quartile cross-classifications of mean daily energy-adjusted micronutrient and fiber intake assessed with Meal-Q, MiniMeal-Q, and the weighed food record (WFR) (N=163).

Nutrients	Same quartile, %		Adjacent quartile, %		Same/adjacent quartile, %		Extreme quartile, %	
	Meal-Q	MiniMeal-Q	Meal-Q	MiniMeal-Q	Meal-Q	MiniMeal-Q	Meal-Q	MiniMeal-Q
Beta-carotene (µg)^a^	41	41	40	42	81	83	4	6
Thiamine (mg)	27	31	44	43	71	74	7	5
Riboflavin (mg)	37	36	37	40	74	76	4	4
Niacin (mg)	36	34	45	45	81	79	4	5
Vitamin B_6_ (mg)	34	31	41	45	75	76	6	6
Folate (µg)	42	40	38	42	80	82	4	4
Vitamin B_12_ (µg)	44	39	34	33	78	72	5	4
Vitamin C (mg)	39	38	46	45	85	83	2	3
Vitamin D (µg)	36	35	40	40	76	75	7	6
Vitamin E (mg)	40	42	34	33	74	75	4	4
Calcium (mg)	36	35	38	36	74	71	7	9
Iron (mg)	38	37	41	40	79	77	4	5
Magnesium (mg)	42	39	40	44	82	83	3	3
Phosphorus (mg)	33	34	42	43	75	77	7	7
Potassium (mg)	36	37	42	42	79	79	5	6
Selenium (µg)	41	38	37	42	78	80	4	6
Sodium (mg)^b^	33	35	36	32	69	67	10	11
Zinc (mg)	34	33	43	43	77	77	7	7
Fiber (g)	53	55	37	34	90	89	1	3

^a^n=162 because of exclusion of 1 participant with implausibly high intake.

^b^n=160 because of exclusion of 3 participants with implausibly high intakes.

**Figure 3:**
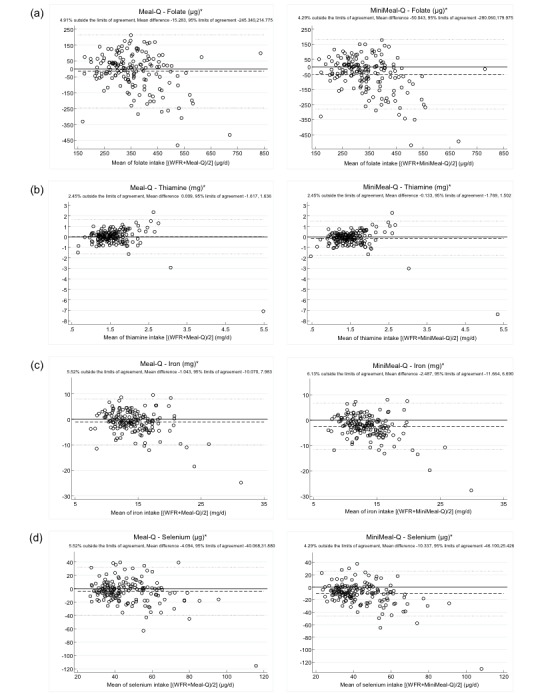
Bland-Altman plots with the weighed food record (WFR), Meal-Q, and MiniMeal-Q for (a) folate, (b) thiamine, (c) iron, and (d) selenium (N=163). Differences in intake between the WFR and the questionnaires are plotted against the mean of the 2 methods. The solid line indicates the reference line of zero difference. The long-dashed line shows the mean difference. The short-dashed lines show the 95% limits of agreement (mean difference ±2 SD). *: Energy-adjusted.

The Bland-Altman plots with the WFR were similar for Meal-Q and MiniMeal-Q as seen in Table 4 and Figure 3 (showing an example of 8 micronutrients) and Multimedia Appendices 1-4. Niacin was largely underestimated by approximately 20 mg for both questionnaires. Most nutrients showed increasing underestimation with increasing intakes, and some also had a trend of increasing variance at higher intakes. In contrast, fiber had a larger variance at lower compared to higher intakes. Most of the nutrients had a varying bias over the intake range (ie, both underestimation and overestimation of intake with a magnitude approximately the same size of the mean intake). However, zinc, magnesium, potassium, and phosphorus showed a less varying bias.

Table 5 shows the Spearman correlation coefficients between Meal-Q, MiniMeal-Q, and the WFR. Correlations for Meal-Q for crude intakes were in the range ρ=.16-.66. Excluding the statistically nonsignificant correlation for sodium, the energy-adjusted correlations for Meal-Q ranged from ρ=.28-.67 and the deattenuated correlations ranged from ρ=.31-.69. The correlations were very similar for MiniMeal-Q, except for thiamine, which showed a stronger correlation with MiniMeal-Q than with Meal-Q.

**Table 4:** Overview of results from Bland-Altman plots^a^ of Meal-Q and MiniMeal-Q in comparison with the weighed food record (WFR) (n=163).

Nutrients	Meal-Q		MiniMeal-Q		Meal-Q and MiniMeal-Q trends^b^
	Mean difference	±2 SD	Mean difference	±2 SD	
Beta-carotene (µg)^c^	427	–4100, 4985	285	–4300, 4873	Increasing variance with increasing intakes
Thiamine (mg)	0.01	–1.6, 16	–0.1	–1.8, 1.5	Increasing variance with increasing intakes
Riboflavin (mg)	–0.2	–1.3, 0.8	–0.3	–1.3, 0.7	Increasing underestimation with increasing intakes
Niacin (mg)	–21	–36, –5	–22	–37, –7	Increasing underestimation with increasing intakes
Vitamin B_6_ (mg)	–0.4	–1.7, 0.9	–0.6	–1.9, 0.7	Increasing underestimation and variance with increasing intakes
Folate (µg)	–15	–245, 215	–50	–280, 180	Increasing underestimation with increasing intakes
Vitamin B_12_ (µg)	–2.0	–7.6, 3.6	–2.5	–8.0, 3.0	Increasing underestimation with increasing intakes
Vitamin C (mg)	–21	–142, 99	–29	–151, 93	Increasing underestimation and variance with increasing intakes
Vitamin D (µg)	–1.3	–9.4, 6.8	–1.6	–9.7, 6.6	Increasing underestimation with increasing intakes
Vitamin E (mg)	–1.4	–9.8, 6.9	–2.0	–10.0, 6.4	Increasing underestimation and variance with increasing intakes
Calcium (mg)	–113	–803, 576	–183	–892, 526	Increasing variance with increasing intakes
Iron (mg)	–1.0	–10.0, 8.0	–2.5	–12.0, 6.7	Increasing underestimation and variance with increasing intakes
Magnesium (mg)	–7.5	–206.0, 191.0	–41	–244, 162	Increasing underestimation with increasing intakes
Phosphorus (mg)	–164	–779, 450	–291	–904, 322	Increasing underestimation with increasing intakes
Potassium (mg)	–315	–1800, 1180	–640	–2200, 878	Increasing underestimation with increasing intakes
Selenium (µg)	–4	–40, 32	–10	–46, 25	Increasing underestimation with increasing intakes
Sodium (mg)^d^	–753	–2700, 1238	–1000	–3000, 922	Increasing underestimation and variance with increasing intakes
Zinc (mg)	–1.0	–6.5, 4.4	–1.9	–7.3, 3.4	Increasing variance with increasing intakes
Fiber (g)	2.0	–16.0, 20.0	–1.5	–20.0, 17.0	Larger variance at lower intakes than at higher intakes

^a^The Bland-Altman plots are shown in Figure 3 and Multimedia Appendices 1-4.

^b^Trends are similar for Meal-Q and MiniMeal-Q.

^c^n=162 because of exclusion of 1 participant with implausibly high intake.

^d^n=160 because of exclusion of 3 participants with implausibly high intakes.

**Table 5:** Spearman rank correlation coefficients (ρ) between Meal-Q, MiniMeal-Q, and the weighed food record (WFR) (N=163).

Nutrients	Crude ρ		Energy-adjusted ρ		Deattenuated ρ (95% CI)	
	Meal-Q	MiniMeal-Q	Meal-Q	MiniMeal-Q	Meal-Q	MiniMeal-Q
Beta-carotene^b^	.51	.51	.46	.46	.51 (.36, .64)	.51 (.36, .64)
Thiamine	.33	.37	.28	.35	.35 (.16, .52)	.43 (.24, .59)
Riboflavin	.16	.15	.39	.38	.42 (.27, .55)	.41 (.26, .54)
Niacin	.39	.37	.43	.42	.47 (.32, .59)	.46 (.31, .59)
Vitamin B_6_	.39	.40	.32	.32	.35 (.19, .49)	.35 (.19, .49)
Folate	.50	.50	.50	.50	.53 (.39, .64)	.53 (.39, .64)
Vitamin B_12_	.39	.28	.46	.37	.51 (.36, .63)	.41 (.26, .55)
Vitamin C	.53	.52	.53	0·50	.57 (.36, .64)	.54 (.41, .65)
Vitamin D	.34	.32	.31	.30	.36 (.19, .50)	.34 (.18, .49)
Vitamin E	.30	.30	.42	.42	.48 (.32, .61)	.48 (.33, .61)
Calcium	.23	.22	.29	.24	.31 (.16, .45)	.25 (.09, .40)
Iron	.44	.43	.42	.38	.46 (.31, .59)	.42 (.27, .55)
Magnesium	.52	.52	.54	.52	.56 (.44, .66)	.54 (.41, .64)
Phosphorus	.36	.37	.36	.36	.39 (.24, .52)	.39 (.24, .52)
Potassium	.42	.42	.41	.38	.43 (.29, .56)	.40 (.25, .52)
Selenium	.32	.30	.42	.41	.45 (.30, .57)	.44 (.30, .57)
Sodium^c^	.32	.32	.15	.12	.16 (-.01, .32)	.14 (-.04, .30)
Zinc	.33	.34	.31	.31	.34 (.18, .49)	.35 (.19, .49)
Fiber	.66	.65	.67	.65	.69 (.60, .77)	.67 (.57, .75)

^a^All correlation coefficients were significant (*P*=.00-.04), except for energy-adjusted sodium (*P*=.06) and deattenuated sodium assessed with Meal-Q as well as crude riboflavin (*P*=.06), energy-adjusted sodium (*P*=.12) and deattenuated sodium assessed with MiniMeal-Q.

^b^n=162 because of exclusion of 1 participant with implausibly high intake.

^c^n=160 because of exclusion of 3 participants with implausibly high intakes.

### Reproducibility

Table 6 shows the absolute intake of micronutrients and fiber assessed with the 2 administered Meal-Q questionnaires in group 2. There were no statistically significant differences between the questionnaires. The proportion of participants classified into the same or adjacent quartile was 86% to 97% and in the extreme quartile 0% to 3% (Table 7). Crude ICCs were in the range of .45-.85 and energy-adjusted ICCs in the range of .50-.76 (Table 7).

**Table 6:** The median and interquartile range (IQR) of daily micronutrient and fiber intake assessed with Meal-Q 1 and Meal-Q 2 from group 2, and the median (IQR) difference in intake between the questionnaires (n=87).

Nutrients	Meal-Q 1		Meal-Q 2^a^		Difference^b^ (Meal-Q 1–Meal-Q 2)	
	Median	IQR	Median	IQR	Median	IQR
Beta-carotene (µg)	3246	2776	2441	3626	126	1271
Thiamine (mg)	1.4	0.7	1.5	0.9	0.01	0.48
Riboflavin (mg)	1.7	0.7	1.6	0.7	0.03	0.69
Niacin (mg)	15	9	16	9	–0.06	4.96
Vitamin B_6_ (mg)	2.0	1.0	1.9	1.0	0.03	0.67
Folate (µg)	320	187	332	218	14	105
Vitamin B_12_ (µg)	3.7	2.1	4.0	2.5	–0.18	1.44
Vitamin C (mg)	108	81	99	93	–0.78	41.10
Vitamin D (µg)	4.9	2.8	5.2	3.6	–0.19	3.32
Vitamin E (mg)	9.6	5.2	9.5	5.6	0.27	3.47
Calcium (mg)	860	449	888	454	8.95	299.27
Iron (mg)	13	8	13	10	–0.07	4.37
Magnesium (mg)	406	212	418	233	10	112
Phosphorus (mg)	1419	668	1483	518	1.92	430.63
Potassium (mg)	3208	1719	3116	1584	38	963
Selenium (µg)	43	23	46	23	–0.12	15.93
Sodium (mg)	2466	984	2499	1294	–75	757
Zinc (mg)	11.0	4.9	11.0	3.7	0.004	3.556
Fiber (g)	28	19	25	22	0.20	8.53

^a^Missing values on Meal-Q 2 for 4 participants.

^b^None were statistically significant using Wilcoxon signed rank test (*P*=.07-.96).

**Table 7:** Quartile cross-classifications of Meal-Q 1 and Meal-Q 2^a^ from group 2, and crude and energy-adjusted intraclass correlation coefficients (ICC) (n=87).

Nutrients	Participants, %				ICC (95% CI)	
	Same quartile	Adjacent quartile	Same/adjacent quartile	Extreme quartile	Crude	Energy-adjusted
Beta-carotene (µg)	53	43	96	1	.85 (.79, .91)	.75 (.66, .84)
Thiamine (mg)	57	34	91	2	.54 (.40, .69)	.64 (.51, .76)
Riboflavin (mg)	59	33	92	3	.45 (.28, .62)	.63 (.51, .76)
Niacin (mg)	52	41	93	0	.66 (.54, .78)	.76 (.67, .85)
Vitamin B_6_ (mg)	53	36	89	1	.49 (.33, .65)	.50 (.34, .66)
Folate (µg)	59	38	97	1	.71 (.60, .81)	.73 (.63, .83)
Vitamin B_12_ (µg)	59	31	90	1	.60 (.47, .74)	.65 (.53, .78)
Vitamin C (mg)	52	43	95	1	.80 (.73, .88)	.74 (.64, .83)
Vitamin D (µg)	43	43	86	3	.56 (.42, .70)	.55 (.41, .70)
Vitamin E (mg)	57	34	91	0	.73 (.64, .83)	.73 (.63, .83)
Calcium (mg)	51	38	89	1	.49 (.33, .65)	.66 (.54, .78)
Iron (mg)	51	41	92	2	.61 (.47, .74)	.61 (.48, .74)
Magnesium (mg)	66	31	97	1	.64 (.51, .76)	.73 (.64, .83)
Phosphorus (mg)	54	33	87	3	.46 (.29, .62)	.62 (.49, .75)
Potassium (mg)	56	38	94	0	.65 (.52, .77)	.80 (.73, .88)
Selenium (µg)	61	33	94	1	.64 (.52, .77)	.72 (.61, .82)
Sodium (mg)	57	38	95	1	.53 (.38, .68)	.59 (.45, .72)
Zinc (mg)	46	40	86	1	.50 (.35, .66)	.63 (.50, .76)
Fiber (g)	55	39	94	0	.77 (.69, .86)	.71 (.61, .82)

^a^Missing values on Meal-Q 2 for 4 participants.

## Discussion

### Principal Results

This validation study suggests Meal-Q and MiniMeal-Q are useful tools for ranking micronutrient and fiber intake in epidemiological studies, with the exception of sodium. Furthermore, Meal-Q’s reproducibility results indicate good assessment reliability.

Regarding assessment of absolute intake, both questionnaires underestimated intake of most micronutrients as compared to the WFR. This underestimation may be partly explained by the methodological differences between the methods. A food record has an open-ended design and is aimed to assess the whole diet during a consecutive number of days. In contrast, a questionnaire has predefined items and frequencies and naturally cannot assess the entire diet. Rather, the aim of a questionnaire is to assess dietary intake in a way that enables ranking of low to high consumers. Because risk comparisons in epidemiological studies commonly are made between different strata of intake, the ranking ability of dietary intake is usually of more interest than assessment of absolute intake [14,20]. Therefore, we conclude that Meal-Q and MiniMeal-Q are useful instruments in an epidemiological setting.

The captured between-person variance in intake assessed with MiniMeal-Q as compared to Meal-Q demonstrated only a minor loss of information when using MiniMeal-Q despite having approximately 30% fewer food items. This indicates MiniMeal-Q is a valuable alternative when a shorter questionnaire is desirable.

Acknowledging that the evaluation of MiniMeal-Q is made with truncated Meal-Q data, comparisons between them should be interpreted carefully. Comparing our results to other validation studies should also be done with caution given that differences in study design and participant demographics may affect the results. Yet, bearing its limits in mind, such comparisons, which are commonly made, are crucial in evaluating a questionnaire’s performance.

### Comparison With Prior Work

The cross-classifications with the WFR showed both questionnaires to yield ranking agreements comparable to or better than other similar validation studies [21-25], of which 2 evaluated Web-based FFQs. The highest-ranking agreement for Meal-Q and MiniMeal-Q was seen for fiber with 89% to 90% placed into the same or adjacent quartile, which is greater than in some other studies [21,23-24]. The lowest ranking agreement was seen for sodium, as has been shown previously [21,23], and which likely reflects the difficulty in assessing salt intake.

The Bland-Altman plots showed that Meal-Q and MiniMeal-Q had difficulties in precision as seen by the large variance. This varying bias over the intake range was also indicated by the limits of agreement, which for some nutrients deviated from 5%. For most nutrients, the questionnaires did not perform as well in assessing high intakes. This might be explained by a limitation of food items, excessive grouping of several food items on each row, lack of high frequency alternatives, or the use of standard portion sizes for many food items. The overall large variance seen for most nutrients could arise from various sources (eg, a limited frequency range of the questionnaires and/or a high between-person variation in the WFR). Although the Bland-Altman method has been recommended for use in validation studies, it should be noted that we would not expect an absolute agreement between the questionnaires and the WFR because of their inherent methodological differences. Instead, the plots are helpful in assessing the magnitude of the inaccuracy and detecting potential varying bias. Despite the varying bias over the intake range seen in the Bland-Altman plots, the cross-classifications of quartiles indicated that both Meal-Q and MiniMeal-Q were able to yield a good ranking ability.

The limited number of studies using Bland-Altman plots for assessment of micronutrient validity and that some of them used log-transformed values makes comparisons with our results difficult. However, 2 other studies have also detected varying bias over the intake range. Labonté et al [23] showed similar results in variance for fiber intake and Pinto et al [25] showed a larger variance for folate and iron intake than seen in the present study.

The energy-adjusted and deattenuated Spearman correlation coefficients in the current study were similar to or better than correlations obtained in other validation studies with comparable study design [21-27]. Sodium showed a statistically nonsignificant correlation with the WFR for both questionnaires, which has also been seen previously [21,23]. Furthermore, in a review of 392 validation studies of vitamin intake, Henríquez-Sánchez et al [28] showed mean correlations between a FFQ and a dietary record in the range *r*=.41-.53. Another review of 109 validation studies of iron, calcium, selenium, and zinc reported mean correlations between a FFQ and a dietary record ranging from *r*=.36-.60 [29]. Both reviews show that the correlations in our study are in-line with other validation studies for most nutrients, with the exception of thiamine, riboflavin, vitamin B_6_, vitamin D, vitamin E, calcium, and zinc, which had correlations somewhat lower in the present study. Only correlations for vitamin B_12_, niacin, riboflavin, vitamin E, calcium, magnesium, selenium, and fiber improved after energy adjustment, a phenomenon also seen previously [25,26]. A possible explanation for this is varying correlation with energy between different nutrients [14], a feature that also depends on the population.

The use of correlation coefficients in validation studies is extensive, but has been criticized because they only measure a relationship and not the agreement between 2 methods [15]. However, as mentioned, we would not expect an absolute agreement between a FFQ and a food record because FFQs are designed to rank individuals rather than to assess absolute intake [20]. In this way, the correlation coefficient is a useful measure of validity because it assesses the ranking ability.

The sodium intake assessed with Meal-Q and MiniMeal-Q only included salt in food items and dishes in the nutrient database. Both questionnaires have a yes/no question regarding salt in cooking and table salt; however, because it is difficult to estimate amounts, this information was not included in the nutrient calculations. The WFR could potentially capture added salt; however, this was only reported for a minor fraction of all food items. Hence, the sodium assessed with the questionnaires and the WFR both originate from salt already present in food items and dishes from the nutrient database. Therefore, the low validity for sodium could best be explained by a general large random variation in assessment between the questionnaire and the WFR.

The reproducibility of Meal-Q indicated that it performed well in its reliability to rank dietary intake, with a high proportion of participants in the same/adjacent quartile and a low proportion of misclassified participants. The quartile cross-classifications were comparable to Labonté et al [23]. Energy-adjusted correlations between repeated FFQs have generally ranged between *r*=.5-.8 in other studies [30] and Meal-Q showed quite similar results. The ICCs for fiber, vitamin E, vitamin B_6_, niacin, vitamin C, beta-carotene, folate, magnesium, and potassium in the present study were, on average, lower than those found by Schröder et al [31]. Furthermore, the ICCs were slightly lower than the Pearson correlations found by Labonté et al [23], yet higher than the Pearson correlations found by Pinto et al [25].

### Limitations and Strengths

A strength of this study was the large sample size for this type of validation study. Moreover, there was low dropout and high compliance for the assessment methods throughout the entire study. The high compliance probably reflects a well-motivated study population, something that is vital for the study’s internal validity. The motivation might arise from a general higher interest in health among self-selected participants as compared to invited participants. Furthermore, participants with nutritional backgrounds might also be more motivated than those without this background. It should be acknowledged that the young and primarily female study population might have implications on external validity. Regarding data handling, the Web-based format of the questionnaires and the WFR minimized potential errors in the conversion of crude consumption data into nutrient intakes. Web-based formats have previously shown to improve data quality [4,32].

In the validation of a dietary assessment method, the reference method should have measurement errors independent from those of the test method. Because the WFR is an open-ended prospective method and an FFQ is a retrospective method with predefined food items and frequencies, dependent measurement errors are less likely to occur. Nevertheless, both methods are susceptible to social desirability, as are all dietary assessment methods. This could affect them in similar ways and increase their error dependency as a result. Also, both methods are linked to the same nutrient database. Therefore, a validation study of a dietary assessment method should be evaluated for relative validity rather than absolute validity. Unfortunately, the present study did not have the means to include an objective reference method for micronutrient intake as biomarkers (eg, urinary potassium, thiamine, and sodium), which would have been a valuable complement to the WFR.

Meal-Q and MiniMeal-Q reflect dietary intake during the past few months, whereas the WFR captures dietary intake over 7 consecutive days; hence, a perfect agreement should not be expected. Ideally, the WFR would have been performed repeatedly over a longer time period to better mirror the assessment aim of the questionnaires. Also, for the reproducibility analysis, the second Meal-Q should have been administered after a slightly longer time period preferably to decrease the influence from the first questionnaire. However, time constraints made a longer validation study impossible. In the comparisons between questionnaires and the WFR, adjustments for within-person variance in the WFR were made to minimize the effect of day-to-day variations in intake. Furthermore, MiniMeal-Q ideally should have been evaluated in a separate validation study; however, this was not possible because of time constraints.

### Conclusions

This validation study demonstrated that Meal-Q and MiniMeal-Q are useful questionnaires for ranking micronutrient and fiber intake in epidemiological studies using Web-based data collection. However, assessment of sodium intake requires further attention in future questionnaire versions. Furthermore, the reproducibility results showed Meal-Q to have good reliability. It should be noted that the study was conducted in a young, primarily female, and well-educated study population, and that MiniMeal-Q merits its own validation study.

## Multimedia Appendix 1

Bland-Altman plots with the WFR, Meal-Q and MiniMeal-Q for (e) beta-carotene (n=162 due to exclusion of one subject with implausibly high intake), (f) riboflavin, (g) niacin and (h) vitamin b6 (n=163). Differences in intake between the WFR and the questionnaires are plotted against the mean of the two methods. The solid line indicates the reference line of zero difference. The long-dashed line shows the mean difference. The short-dashed lines show the 95% limits of agreement (mean difference ±2 SD). * Energy-adjusted.

## Multimedia Appendix 2

Bland-Altman plots with the WFR, Meal-Q and MiniMeal-Q for (i) vitamin B12, (j) vitamin C, (k) vitamin D and (l) vitamin E (n=163). Differences in intake between the WFR and the questionnaires are plotted against the mean of the two methods. The solid line indicates the reference line of zero difference.The long-dashed line shows the mean difference. The short-dashed lines show the 95% limits of agreement (mean difference ±2 SD). * Energy-adjusted.

## Multimedia Appendix 3

Bland-Altman plots with the WFR, Meal-Q and MiniMeal-Q for (m) calcium, (n) magnesium, (o) phosphorus and (p) potassium (n=163). Differences in intake between the WFR and the questionnaires are plotted against the mean of the two methods. The solid line indicates the reference line of zero difference. The long-dashed line shows the mean difference. The short-dashed lines show the 95% limits of agreement (mean difference ±2 SD). * Energy-adjusted.

## Multimedia Appendix 4

Bland-Altman plots with the WFR, Meal-Q and MiniMeal-Q for (q) sodium (n=160 due to exclusion of three subjects with implausibly high intakes), (r) zinc and (s) fiber (n=163). Differences in intake between the WFR and the questionnaires are plotted against the mean of the two methods. The solid line indicates the reference line of zero difference. The long-dashed line shows the mean difference. The short-dashed lines show the 95% limits of agreement (mean difference ±2 SD). * Energy-adjusted.

## Abbreviations

BMI: body mass index
DLW: doubly labeled water
FFQ: food frequency questionnaire
ICC: intraclass correlation coefficient
PAL: physical activity level
WFR: weighed food record
