# Supplementary figures and images for: Relative Validity of Micronutrient and Fiber Intake Assessed With Two New Interactive Meal- and Web-Based Food Frequency Questionnaires
Source: J Med Internet Res. 2014 Feb 21;16(2):e59. doi: 10.2196/jmir.2965 (PMC3961697; doi:10.2196/jmir.2965)

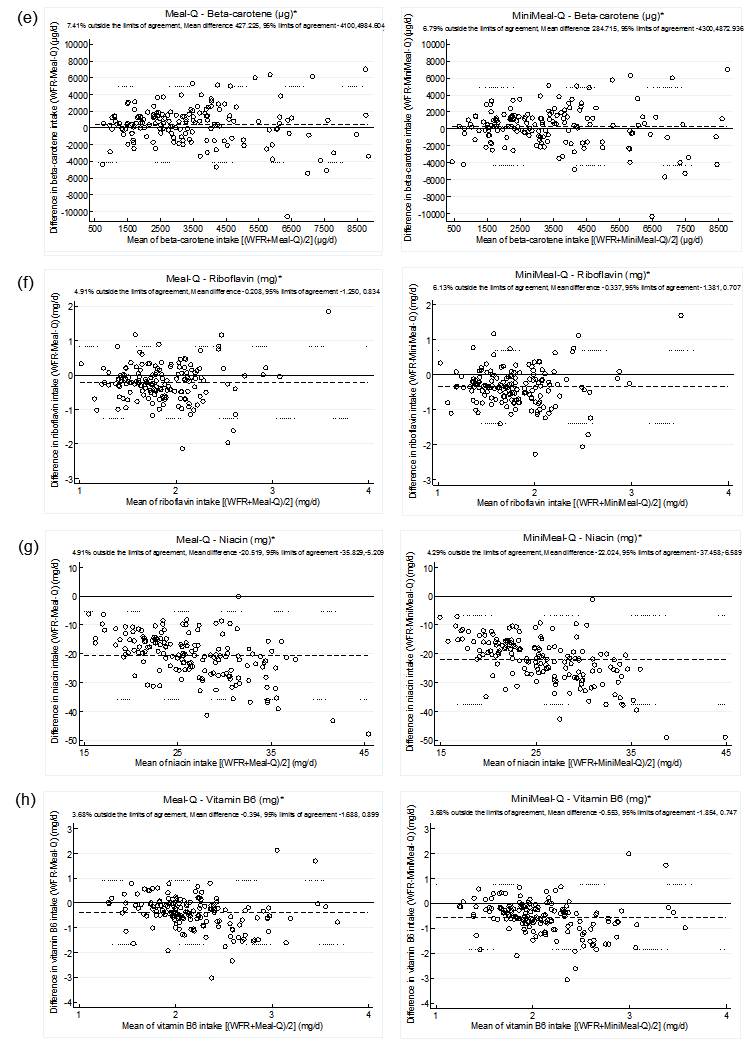

Supplement: Supplementary file 1 [file jmir_v16i2e59_app1.jpg]

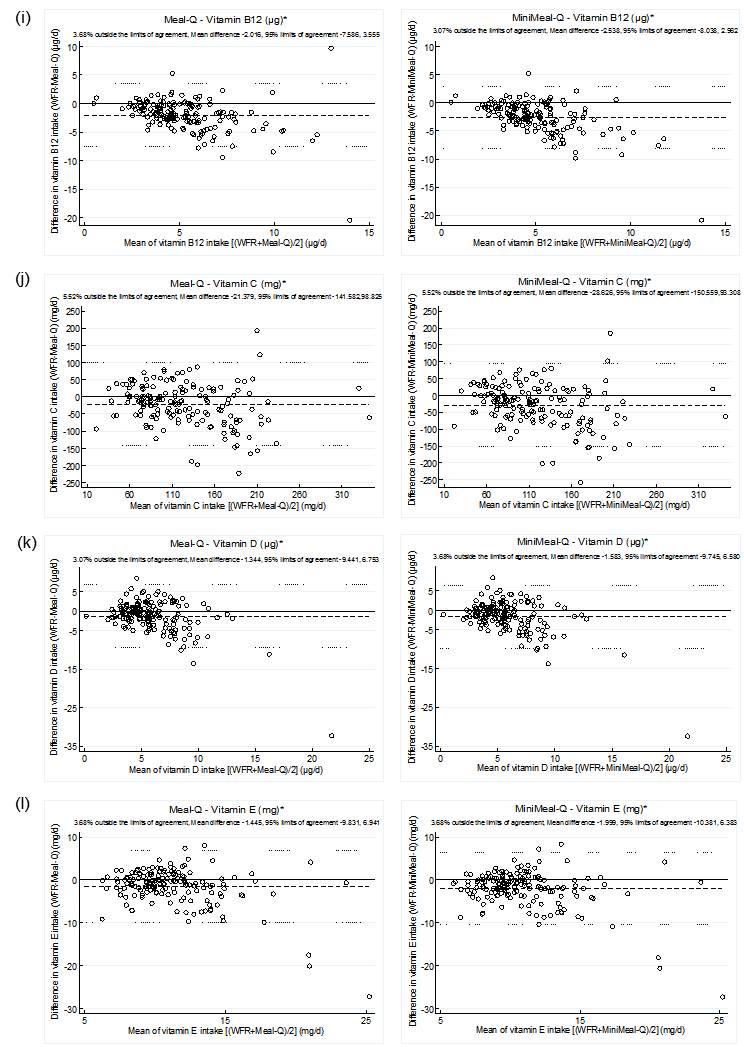

Supplement: Supplementary file 2 [file jmir_v16i2e59_app2.jpg]

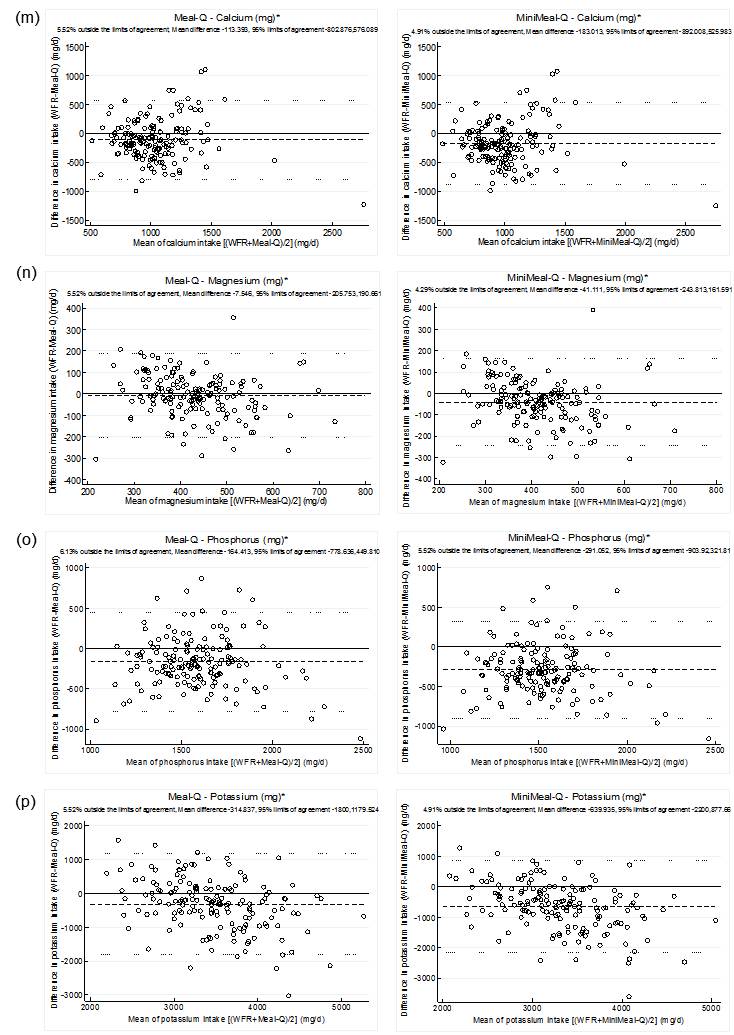

Supplement: Supplementary file 3 [file jmir_v16i2e59_app3.jpg]

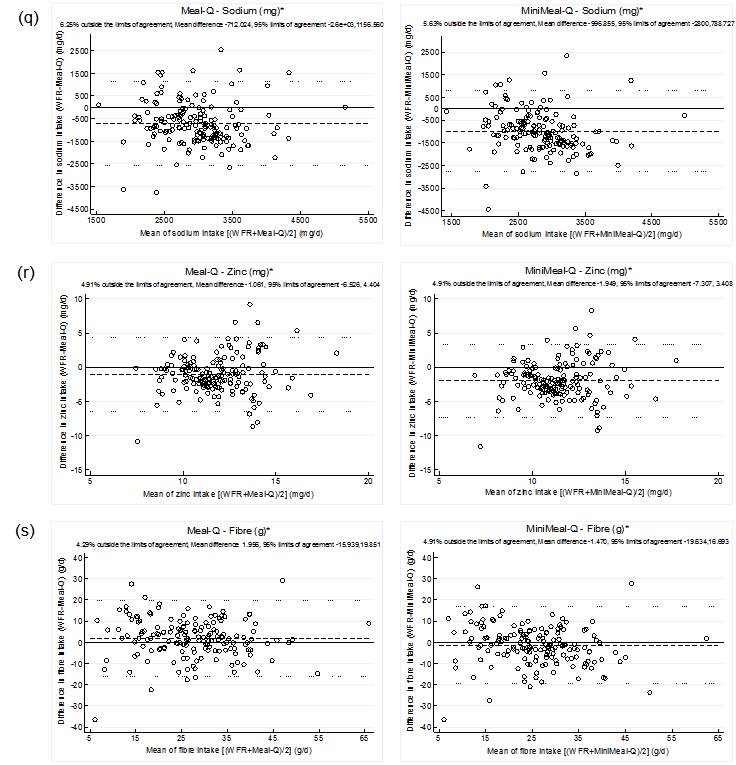

Supplement: Supplementary file 4 [file jmir_v16i2e59_app4.jpg]
